# Comparative analysis of swine leukocyte antigen gene diversity in European farmed pigs

**DOI:** 10.1111/age.13090

**Published:** 2021-05-24

**Authors:** S. E. Hammer, T. Duckova, S. Groiss, M. Stadler, M. Jensen‐Waern, W. T. Golde, U. Gimsa, A. Saalmueller

**Affiliations:** ^1^ Department of Pathobiology Institute of Immunology University of Veterinary Medicine Vienna Veterinaerplatz 1 Vienna 1210 Austria; ^2^ Department of Clinical Sciences Swedish University of Agricultural Sciences PO Box 7054 Uppsala 750 07 Sweden; ^3^ Moredun Research Institute Edinburgh EH26 OPZ UK; ^4^ Institute of Behavioural Physiology Leibniz Institute for Farm Animal Biology Wilhelm‐Stahl‐Allee 2 Dummerstorf 18196 Germany

**Keywords:** polymorphism, sequence‐specific primers PCR, *Sus scrofa*, swine leukocyte antigen

## Abstract

In Europe, swine represent economically important farm animals and furthermore have become a preferred preclinical large animal model for biomedical studies, transplantation and regenerative medicine research. The need for typing of the swine leukocyte antigen (SLA) is increasing with the expanded use of pigs as models for human diseases and organ‐transplantation experiments and their use in infection studies and for design of veterinary vaccines. In this study, we characterised the SLA class I (*SLA‐1, SLA‐2, SLA‐3*) and class II (*DRB1, DQB1, DQA*) genes of 549 farmed pigs representing nine commercial pig lines by low‐resolution (Lr) SLA haplotyping. In total, 50 class I and 37 class II haplotypes were identified in the studied cohort. The most common SLA class I haplotypes Lr‐04.0 (SLA‐1*04XX‐SLA‐3*04XX(04:04)‐SLA‐2*04XX) and Lr‐32.0 (SLA‐1*07XX‐SLA‐3*04XX(04:04)‐SLA‐2*02XX) occurred at frequencies of 11.02 and 8.20% respectively. For SLA class II, the most prevalent haplotypes Lr‐0.15b (DRB1*04XX(04:05/04:06)‐DQB1*02XX(02:02)‐DQA*02XX) and Lr‐0.12 (DRB1*06XX‐DQB1*07XX‐DQA*01XX) occurred at frequencies of 14.37 and 12.46% respectively. Meanwhile, our laboratory has contributed to several vaccine correlation studies (e.g. Porcine Reproductive and Respiratory Syndrome Virus, Classical Swine Fever Virus, Foot‐and‐Mouth Disease Virus and Swine Influenza A Virus) elucidating the immunodominance in the T‐cell response with antigen specificity dependent on certain SLA‐I and SLA‐II haplotypes. Moreover, these SLA–immune response correlations could facilitate tailored vaccine development, as SLA‐I Lr‐04.0 and Lr‐32.0 as well as SLA‐II Lr‐0.15b and Lr‐0.12 are highly abundant haplotypes in European farmed pigs.

The porcine major histocompatibility complex (MHC) harbours the highly polymorphic swine leukocyte antigen (SLA) class I and II gene clusters encoding glycoproteins which present antigenic peptides to T cells that are required to stimulate the adaptive immune response (Lunney *et al*. 2009; Hammer *et al*. 2020; Kamal *et al*. 2020). As pathogen effects on SLA gene expression drive swine immune responses, the SLA complex plays a key role for swine models in biomedical research (reviewed in Hammer *et al*. 2020). Associations of SLA class I and/or class II genes or haplotypes with differences in swine vaccine and disease responses are well documented (reviewed in Lunney *et al*. 2009). In vaccine research, either genetically defined pig lines (e.g., Babraham pigs) or outbred pig lines are used (Tungatt *et al*. 2018; De León *et al*. 2020). As well as using SLA‐typed animals in vaccine research, pigs are often used to develop disease models and for basic research studying allogeneic and xenogeneic transplantation (reviewed in Ladowski *et al*. 2019; Hammer *et al*. 2020; Ladowski *et al*. 2021). To understand and control SLA complexity, mainly miniature swine models are used to establish SLA‐inbred/‐defined pig lines (reviewed in Hammer *et al*. 2020; Ladowski *et al*. 2021). In contrast, in vascularised composite allograft transplantation or for end‐stage renal disease, porcine transplantation models have been established with SLA‐mismatched outbred pigs (I. Arenas Hoyos *et al*. and M. Jensen‐Waern *et al*. unpublished data).

Here we propose two underlying rationales for conducting SLA haplotyping‐assisted animal trials in vaccine and transplantation research: (i) SLA typing of the resource population enables directed mating of founder animals based on their SLA‐background (Fig. S1): and (ii) the designation of SLA‐defined study groups achieves an experimental advantage of pre‐selecting animals expressing certain SLA phenotypes and thus enhancing the understanding of experimental outcomes (Fig. S1). As a prerequisite for transplantation and vaccine research, our laboratory provides information about the MHC background usingy high‐throughput low‐resolution (Lr) SLA haplotyping in swine specifying SLA gene‐specific allele groups (reviewed in Hammer *et al*. 2020).

We have contributed to several correlation studies addressing vaccine design against Porcine Reproductive and Respiratory Syndrome Virus (PRRSV), Classical Swine Fever Virus, Foot‐and‐Mouth Disease Virus (FMDV) and Swine Influenza A Virus (FLUAVsw) by SLA haplotyping outbred pigs. Furthermore, our laboratory is involved in studies with minipigs for various purposes in transplantation research (Fig. 1, Table S1). In this study, we present comprehensive data about SLA alleles and low‐resolution haplotypes and their prevalence in nine commercial European pig populations.

**Figure 1:**
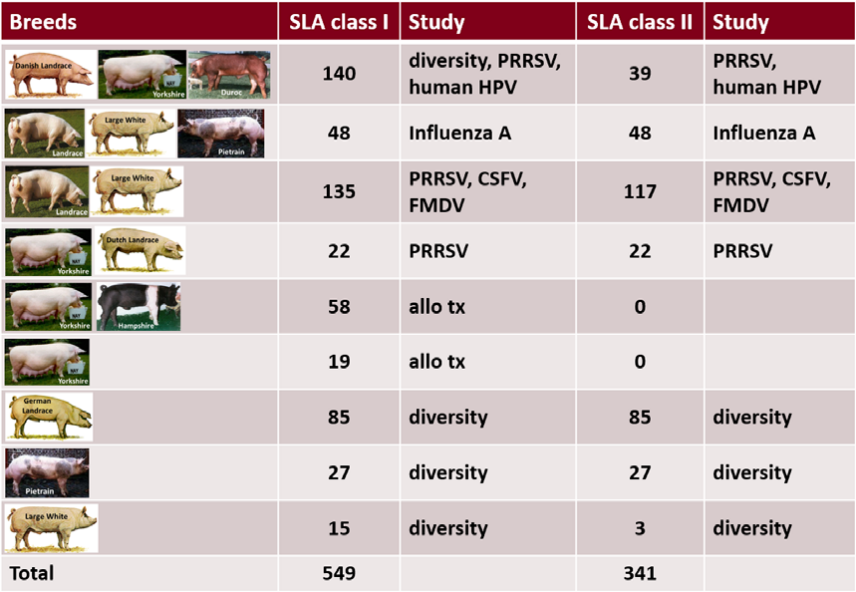
List of European farmed pigs incorporated in the present study. CSFV, Classical Swine Fever Virus; FMDV, Foot‐and‐Mouth Disease Virus; HPV, Human Papilloma Virus; PRRSV, Porcine Respiratory and Reproductive Syndrome Virus; tx, transplantation.

A total of 549 farmed pigs (Fig. 1, Table S1) representing nine commercial pig lines were genotyped for their SLA class I and II haplotypes by running low‐resolution PCR screening assays on Peripheral blood mononuclear cell (PBMC)‐ or whole blood‐derived genomic DNA. Therefore, genomic DNA was isolated from 5 × 10^6^ porcine PBMCs or 200 µl whole blood using commercial kits following the manufacturer’s instructions (DNeasy Blood and Tissue Kit, Qiagen; E.Z.N.A.^®^ Blood and Tissue DNA Kit, Omega Bio‐tek, Inc.). SLA class I (SLA‐I) and SLA class II (SLA‐II) low‐resolution haplotypes (Lr‐Hp) were identified by a PCR‐based typing assay to define the animals’ MHC backgrounds on the allele‐group level. SLA typing was performed by PCR with the complete set of typing primers specific for the allele groups of three SLA‐I loci (*SLA‐1*, *SLA‐2* and *SLA‐3*) and three SLA‐II loci (*DRB1*, *DQB1* and *DQA*) (Table S2; Ho *et al*. 2009a, 2010; Essler *et al*. 2013; Gimsa *et al*. 2017). The criteria and nomenclature used for SLA‐I and SLA‐II haplotyping were based on those proposed by the SLA Nomenclature Committee (Ho *et al*. 2009b and reviewed in Hammer *et al*. 2020). Interpretation of the results was deduced from the presence of allele‐specific PCR products of the expected size in each lane. Low‐resolution SLA‐I and ‐II haplotypes were assigned based on comparison with previously published haplotypes (Ho *et al*. 2009a, 2010; Gao *et al*. 2017, reviewed in Hammer *et al*. 2020) and unpublished breed‐ or farm‐specific haplotypes (C.‐S. Ho *et al*. unpublished data).

The studied cohort of 549 farmed pigs representing nine commercial pig lines comprised 50 SLA‐I Lr‐Hp, including three potential novel allele‐group combinations (Lr‐01.0/04.0, Lr‐V.0, Lr‐Y1.0) (Table 1). Eight haplotypes (Lr‐04.0, Lr‐32.0, Lr‐22.0, Lr‐01.0, Lr‐59.0, Lr‐24.0, Lr‐37.0 and Lr‐43.0) explained 51.37% of the SLA‐I diversity (Figs S2a & S3a). The two most abundant SLA‐I haplotypes – Lr‐04.0 (SLA‐1*04XX‐SLA‐3*04XX(04:04)‐SLA‐2*04XX) and Lr‐32.0 (SLA‐1*07XX‐SLA‐3*04XX(04:04)‐SLA‐2*02XX) – occurred at frequencies of 11.02 and 8.20% respectively (Figs. S2a & S3a). Note: ‘XX’ indicates SLA gene‐specific allele groups. Comparing these findings with previously published SLA‐typing studies, Lr‐04.0 was also found in the pig populations (i) of studies from the Kansas State University (KSU, PRRSV study, unknown breed raised in the USA), (ii) of studies with Porcine Circo Virus (PCV, pigs with susceptibility to subgroups of PCV type 2, unknown breed raised in the USA), (iii) of the Big Pig group (Large White/Landrace crosses raised in the USA) and (iv) of Yorkshire pigs of Canadian origin (Ho *et al*. 2009a; Gao *et al*. 2017). In contrast, Lr‐32.0 was observed only in the pig groups Big Pig and Landrace of Canadian origin (Ho *et al*. 2009a; Gao *et al*. 2017). Lr‐22.0 and Lr‐01.0 were shared with KSU, PCV and Big Pig, and the latter with the Yorkshire only (Ho *et al*. 2009a; Gao *et al*. 2017). Lr‐59.0 was only found within the PCV group, Lr‐43.0 was found in the KSU group and Lr‐37.0 was shared in Yorkshire pigs, but Lr‐24.0 did not occur in any of these five studied cohorts (Ho *et al*. 2009a; Gao *et al*. 2017).

**Table 1:** Swine leukocyte antigen (SLA) class I low‐resolution haplotypes characterised in nine European commercial pig populations by PCR screening assays

Low resolution haplotype	Allele specificity^1^			Haplotype frequency (%)									
	*SLA‐1*	*SLA‐3*	*SLA‐2*	LRYS/D	LWLR/P	LR × LW	YS × NL LR	YS × Ham	YS	GER LR	P	LW	Combined
				140	48	135	22	58	19	85	27	15	549
01.0/04.0^2^	04XX	01XX	01XX			1.11	13.64						0.82
01.0	01XX	01XX	01XX	3.57	10.42	13.70	15.91			0.59	5.56		6.28
02.0	02XX,07XX	04XX^3^	02XX	8.57						0.59			2.28
04.0	04XX	04XX (04:04)	04XX	18.93	7.29	8.89	6.82	15.79	21.55	1.76			11.02
05.0	04XX	05XX	08XX	1.79	3.13							6.67	0.91
06.0	08XX	06XX (06:01)	05XX	1.79	2.08	2.22				11.76			3.01
07.0	08XX	07XX	05XX	7.14	2.08	1.11				1.76			2.55
08.0	02XX,04XX	03XX	07XX		1.04								0.09
11.0	01XX,09XX	07XX	05XX			0.37					1.85		0.18
16.0 mod^4^	11XX	06XX	09XX			0.74							0.18
18.0	04XX	03XX	06XX		1.04		9.09						0.46
21.0	07:03	06XX (06:01)	05XX			0.74		2.63	4.31				0.73
22.0	08XX	06XX (06:01)	12XX	8.21	1.04	17.04	2.27		2.59				6.74
23.0	12XX	03XX	Blank		2.08								0.18
24.0	Blank^5^	04XX (04:04)	06XX	2.14	11.46	1.48	11.36	2.63	5.17	1.76	16.67	33.33	5.01
25.0	11XX	03XX	07XX	1.07	4.17	0.37				5.88	7.41		2.00
26.0	08XX	05XX	10XX	3.57	2.08			2.63	2.59	0.59	9.26		2.00
27.0	06XX,08XX	01XX	01XX									3.33	0.09
28.0	09XX,15XX	07XX	05XX		13.54	1.11				1.18	3.70		1.82
29.0	Blank	05XX	09XX	1.43	1.04	1.85					1.85		1.00
32.0	07XX	04XX (04:04)	02XX	15.36	7.29	0.74		2.63	21.55	6.47	1.85		8.20
33.0	Blank^5^	05XX	06XX			0.37				1.18			0.27
34.0	Blank	04XX (04:04)	05XX	0.36	1.04	1.85	6.82			11.18			2.64
35.0	12XX,13XX (13:01)	05XX	10XX	0.71	3.13	3.33	11.36	2.63	5.17	4.71	7.41		3.46
36.0	02XX	01XX	11XX		1.04	0.74		7.89	8.62				1.46
37.0	07XX	05XX	09XX			15.56	6.82						4.10
38.0	15XX	04XX (04:04)	12XX + 11:04	1.07	3.13	2.59		13.16		1.76			1.91
39.0	Blank	05XX	10XX	2.86	1.04	0.37		2.63	4.31	3.53	1.85	13.33	2.46
40.0	16XX	05XX	10XX	0.71	1.04						1.85		0.36
42.0	08XX	06XX (06:02)	09XX	0.36									0.09
43.0	11XX	04XX (04:04)	04XX		8.33	0.37	9.09	13.16		5.29	20.37	13.33	3.83
45.0	08XX + 17:01	07XX	08XX + 10XX	6.07	1.04	1.85				4.12			2.73
46.0	12XX	04XX (04:04)	06XX		1.04	0.37				0.59			0.27
47.0	Blank	06XX (06:01)	05XX	1.79		0.37				1.76			0.82
49.0	08XX	05XX	Blank	3.57						4.12			1.55
52.0	Blank	07XX	03XX/11:04							10.00			1.55
53.0	Blank	08XX	11:04/15XX			0.37							0.09
55.0	15XX	04XX (04:04)	11:04			1.85					11.11	23.33	1.73
56.0	11XX	03XX	15XX							1.18			0.18
57.0	02XX	01XX	11XX	3.21		0.74							1.00
58.0	08XX	03XX (03:06)	09XX (09:03)			0.37							0.09
59.0	11XX (11:03)	05XX	16:02		3.13	14.44	2.27	26.32	11.21	1.18			6.19
61.0	07:05	03XX	06XX	5.00						4.12			1.91
62.0	14XX	04:04	06XX	0.71		0.74	2.27			11.76			2.28
64.0	14XX	05XX	10XX								7.41		0.36
66.0	15XX	04XX (04:04)	04XX		3.13		2.27						0.36
67.0	15XX	05XX	10XX		2.08						1.85		0.27
31.0/63.0	15XX	07XX	16XX			1.85							0.46
V.0^4^	Blank	07XX	08XX + 16XX						8.62				0.91
Y1.0^4^	08XX	04XX (04:04) or blank	09XX					2.63	4.31				0.55
n.d.	XXXX	XXXX	XXXX		1.04	0.37		5.26		1.18		6.67	0.55
			No of Lr‐Hp	24	28	33	13	13	12	26	16	7	51

D, Duroc; GER LR, German Landrace; Ham, Hampshire; LR, Landrace; LW, Large White; NL LR, Dutch Landrace; P, Pietrain; YS, Yorkshire; n.d., not defined.

^1^ Allele designations in parentheses indicates medium‐ or high‐resolution specificities.

^2^ Not yet confirmed haplotype.

^3^ Probably owing to the presence of SLA‐3*04XX‐like pseudogenes as this haplotype did not appear to possess an expressed *SLA‐3* gene (Ho *et al*. 2009a).

^4^ Ambiguity could not be resolved owing to the detection of this haplotype in only one heterozygous animal.

^5^ Untyped SLA class I locus.

With respect to the allele groups discovered, SLA‐1 was more polymorphic than SLA‐2 followed by SLA‐3 (Fig. 2). For SLA‐1, we found 23 allele groups, and three of them explained 46.27% of the diversity. In detail, SLA‐1*08XX, SLA‐1*07XX and SLA‐1*blank represented frequencies of 16.58, 15.03 and 14.66% (Fig. 2). Note: ‘Blank’ indicates alleles that cannot be detected with the primer sets utilised in the current study. For SLA‐2, three out of 22 detected allele groups were responsible for 47.63% of the diversity. More precisely, SLA‐2*04XX, SLA‐2*05XX and SLA‐2*02XX showed frequencies of 15.21, 11.75 and 10.84% (Fig. 2). The lesser polymorphic locus, SLA‐3, was characterised by 10 allele groups, and among them, SLA‐3*04XX (39.80%) and SLA‐3*05XX (22.77%) explained 62.57% of the diversity (Fig. 2).

**Figure 2:**
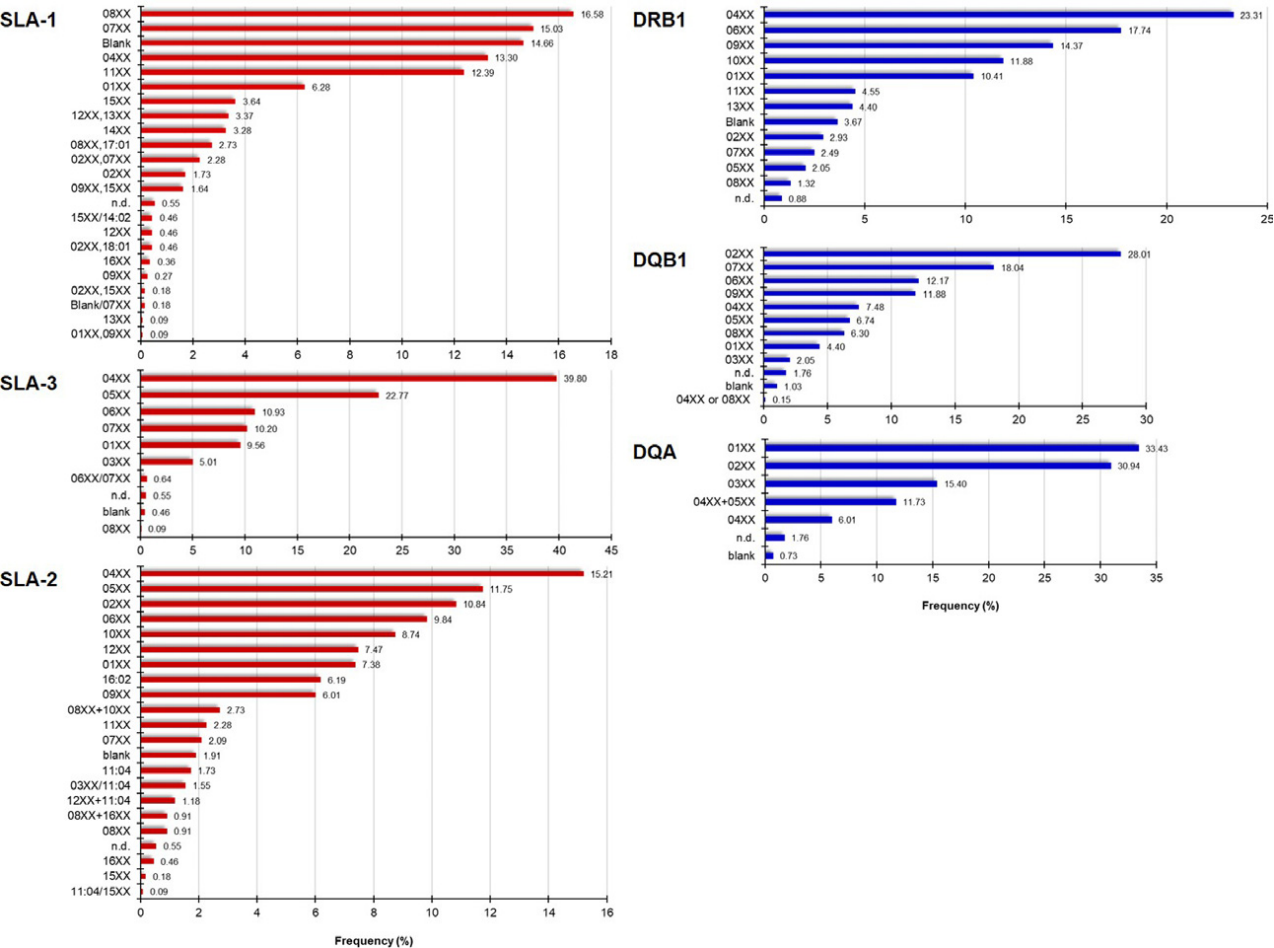
Frequencies (*x*‐axis) of swine leukocyte antigen (SLA) class I (SLA‐1, SLA‐3 and SLA‐2) and class II (DRB1, DQB1 and DQA) allele groups (*y*‐axis) identified in the studied European farmed pigs. ‘Blank’ indicates alleles that cannot be detected with the primer sets utilised in the current study. n.d., Not determined.

For SLA‐II, 37 haplotypes were found, including seven potential novel allele‐group combinations (Lr‐YDLR‐0.1, Lr‐YDLR‐0.2, Lr‐PIE‐0.1, Lr‐PIE‐0.2, Lr‐LWLR‐0.1, Lr‐LRYD‐0.1 and Lr‐NN; Table 2). The four haplotypes Lr‐0.15b, Lr‐0.12, Lr‐0.23 and Lr‐0.21 explained 44.43% of the SLA‐II diversity (Figs S2b & S3b).

**Table 2:** Swine leukocyte antigen class II low‐resolution haplotypes characterised in seven European commercial pig populations by PCR screening assays

Low resolution haplotype	Allele specificity^1^			Haplotype frequency (%)							
	*DRB1*	*DQB1*	*DQA*	LRYS/D	LWLR/P	LR × LW	YS × NL LR	GER LR	P	LW	Combined
				39	48	117	22	85	27	3	341
0.01	01XX	01XX	01XX	3.85	3.13	7.69	9.09		5.56		4.55
0.02	02XX	02XX	02XX	5.13		0.85	4.55			33.33	1.47
0.04	02XX	04XX	02XX			2.56					0.88
0.05	05XX	02XX	02XX	1.28		2.14					0.88
0.06	05XX	08XX	01XX		1.04	2.14	2.27		1.85		1.17
0.07	06XX	06XX	01XX			0.43	4.55				0.44
0.08b^2^	08XX	02XX (02:03)	02XX	2.56	3.13			2.35			1.32
0.09	02XX	04XX	03XX		4.17						0.59
0.10	04XX	08XX	03XX		1.04						0.15
0.11	09XX/09:06	04XX	03XX	1.28	11.46	2.56	4.55	2.94			3.67
0.12	06XX	07XX	01XX	2.56		3.42	9.09	39.41	7.41		12.46
0.13	04XX (04:03)	03XX (03:03)	02XX			0.43			7.41		0.73
0.14	09XX (09:06)	08XX	03XX	1.28	1.04		13.64	6.47	25.93	16.67	4.99
0.15a^2^	04XX (04:01)	02XX	02XX	3.85		3.85		0.59	5.56		2.35
0.15b^2^	04XX (04:05/04:06)	02XX(02:02)	02XX	7.69	19.79	31.20					14.37
0.19a^2^	04XX (04:03/04:04)	07XX	03XX	8.97	4.17	0.43		3.53	16.67		3.96
0.19b^2^	04XX (04:05/04:06)	07XX	03XX				2.27	5.88			1.61
0.20	06XX	03XX	01XX		6.25				3.70		1.17
0.21	01XX	05XX	04XX+05XX^3^	2.56		3.85	18.18	12.94			6.01
0.22	06XX	02XX (02:04)	02XX		1.04	1.28	2.27				0.73
0.23	10XX (10:06)	06XX (06:03)	01XX	10.26	25.00	7.26	18.18	8.82	9.26	33.33	11.58
0.24	07XX	02XX	02XX	6.41	1.04	4.27			1.85		2.49
0.25	13XX	09XX	04XX + 05XX^3^	12.82	3.13	2.14		5.88	11.11		4.99
0.26	11XX	04XX	02XX		3.13	1.71	4.55	4.12			2.35
0.27	09XX/09:06	09XX	04XX + 05XX^3^			16.24				16.67	5.72
0.29	Blank^4^	09XX	04XX + 05XX^3^		1.04			5.29			1.47
0.30	11XX (11:01)	05XX	02XX			0.85	4.55	0.59			0.73
0.32	06XX	Blank	02XX		5.21	0.85					1.03
0.33	11XX (11:01/11:03)	02XX (02:06)	02XX	5.13	4.17	0.85					1.47
0.35	01XX (01:04)	04XX	02XX		1.04						0.15
YDLR‐0.1^5^	06XX	05XX	03XX				2.27				0.15
YDLR‐0.2^5^	06XX	02:02/02:04	03XX			0.43					0.15
PIE‐0.1^5^	01XX	05XX	Blank						1.85		0.15
PIE‐0.2^5^	06XX	03XX	03XX						1.85		0.15
LWLR‐0.1^5^	Blank	02XX	02XX			0.85					0.29
LRYD‐0.1^5^	06XX	02XX	01XX	12.82							1.47
NN^5^	Blank	02XX	02XX	11.54							1.32
n.d.	XXXX	XXXX	XXXX			1.71		1.18			0.88
			No of Lr‐Hp	17	19	25	14	14	13	4	38

D, Duroc; GER LR, German Landrace; Ham, Hampshire; LR, Landrace; LRYD, Landrace/Yorkshire/Duroc crosses; LW, Large White; LWLR, Large White/Landrace crosses; NL LR, Dutch Landrace; P, Pietrain; PIE, Pietrain (Austria); YDLR, Yorkshire/Dutch Landrace crosses; YS, Yorkshire; n.d., not defined.

^1^ Allele designations in parentheses indicates medium‐ or high‐resolution specificities.

^2^ The alphabetical suffix in haplotype designations was used to differentiate between closely related haplotypes (i.e. haplotypes with identical low‐resolution group specificities, but different allele specificities).

^3^ Positive with both DQA*04XX primer sets in lanes D12 and C12 (Table S2b).

^4^ Untyped swine leukocyte antigen class II locus.

^5^ Not yet confirmed haplotype.

The two most abundant SLA‐II haplotypes, Lr‐0.15b (DRB1*04XX(04:05/04:06)‐DQB1*02XX(02:02)‐DQA*02XX) and Lr‐0.12 (DRB1*06XX‐DQB1*07XX‐DQA*01XX), occurred at frequencies of 14.37 and 12.46% respectively (Figs S2b & S3b). With respect to previous studies, Lr‐0.15b was also found in the pig populations KSU, PCV, Big Pig and Yorkshire (Ho *et al*. 2010; Gao *et al*. 2017). Lr‐0.12 and Lr‐0.23 were shared in Big Pig and Landrace together with PCV (Lr‐0.12) and Yorkshire (Lr‐0.23) (Ho *et al*. 2010; Gao *et al*. 2017). In contrast, Lr‐0.21 was observed only in the pig groups KSU and PCV (Ho *et al*. 2010).

As expected, regarding the detected number of SLA‐II allele groups, DRB1 was more polymorphic than DQB1 followed by DQA (Fig. 2). For DRB1, we found 13 allele groups, and two of them explained 41.06% of the diversity. Specifically, DRB1*04XX and DRB1*06XX represented frequencies of 23.31 and 17.74% respectively (Fig. 2). For DQB1, two out of 12 detected allele groups were responsible for 46.04% of the diversity with DQB1*02XX and DQB1*07XX showing frequencies of 28.01 and 18.04% (Fig. 2). The lesser polymorphic locus, DQA, was characterised by seven allele groups and among them DQA*02XX (33.43%) and DQA*07XX (30.94%) explained 64.37% of the diversity (Fig. 2).

In veterinary vaccine design, the characterisation of the peptide‐binding specificity of SLA‐I and SLA‐II molecules is pivotal to understanding adaptive immune responses of swine towards infectious pathogens (reviewed in Hammer *et al*. 2020). Herein we briefly discuss key findings on the correlation of SLA haplotypes and immune responses for the animals enrolled in this study. Immunity against the PRRSV is not well understood, although there is evidence suggesting that virus‐specific T‐cell IFN‐*γ* responses play an important role. It was demonstrated that PRRSV‐vaccinated and challenged pigs carrying SLA‐I haplotype Lr‐01.0/04.0 or Lr‐59.0 and SLA‐II haplotype Lr‐0.27 showed significant IFN‐*γ* responses, pointing towards a positive correlation of SLA haplotype and T‐cell response (Burgara‐Estrella *et al*. 2013). Another PRRSV study suggested that the antigenic region NSP5_156–167_ could be restricted by the SLA‐I haplotype Lr‐22.0, meaning that a T cell will only respond to this particular antigen when it is bound to either SLA‐1*08XX, SLA‐3*06:01 or SLA‐2*12XX. Additionally, pigs demonstrating CD4^+^ T cell responses to the antigenic peptide M_29–43_ were haploidentical, sharing both SLA‐II haplotypes Lr‐0.01 and Lr‐0.15b. This combination appearing exclusively in these animals suggests restriction by one of these two haplotypes (Mokhtar *et al*. 2014, 2016).

A proteome‐wide screening revealed immunodominance in the CD8 T‐cell response against Classical Swine Fever Virus with antigen specificity dependent on SLA‐I haplotypes. The variability in the antigen‐specificity of these immunodominant CD8 T‐cell responses was confirmed to be associated with the expression of distinct SLA‐I haplotypes. Moreover, recognition of NS2_1223–1230_ STVTGIFL (Lr‐22.0) and NS3_1902–1912_ VEYSFIFLDEY (Lr‐01.0) by a larger group of C‐strain vaccinated animals showed that these peptides could be restricted by additional haplotypes (Franzoni *et al*. 2013).

In the analysis of FLUAVsw, the porcine T‐cell response has been poorly characterised to date. In a cohort of 40 outbred pigs, Talker and co‐workers showed that animals with a strong expansion of Ki‐67^+^CD8β^+^ T cells and the highest frequencies of FLUAVsw‐specific cytokine‐producing CD4^+^ T cells were homozygous for the SLA‐I haplotype Lr‐01.0 and for the SLA‐DQA locus (DQA*02XX) (Talker *et al*. 2015, 2016). In 2018, Schwartz and co‐workers fully characterised the SLA background of the inbred Babraham pigs at a high‐resolution level: SLA‐1*14:02‐SLA‐3*04XX‐SLA‐2*11:04 and DRB1*05:01‐DQB1*08:01/02‐DQA*01:03. Based on this SLA‐defined pig model, it was then possible to develop a toolset that included the identification of novel immunodominant FLUAVsw‐derived T‐cell epitopes (Schwartz *et al*. 2018; Tungatt *et al*. 2018).

Previous studies showed the promising potential of dendrimer peptides as vaccine candidates against FMDV. Several B‐cell epitope dendrimers, harbouring a major FMDV antigenic B‐cell site in VP1 protein that is covalently linked to heterotypic T‐cell epitopes from 3A and/or 3D proteins, elicited consistent levels of neutralising antibodies and IFN‐*γ*‐producing cells in pigs (De León *et al*. 2020). Robust correlations of certain SLA haplotypes (Lr‐22.0, Lr‐59.0, Lr‐0.15b, Lr‐0.24 and Lr‐0.27) with antibody titres and IFN‐*γ*‐producing cells support the contribution of SLA class‐II restricted T‐cells to the magnitude of the T‐cell response and to the antibody response evoked by the B_2_T dendrimers, being of potential value for peptide vaccine design against FMDV (De León *et al*. 2020). In addition, Patch and colleagues used inbred minipigs to show that FMDV infection results in induction of cytotoxic T cell responses that are classically antigen specific and MHC restricted (Patch *et al*. 2014). Following on, these investigators used SLA‐1*04:01 and SLA‐2*04:01 class I tetramers to show that, upon vaccination with replication defective adenovirus 5 vectors expressing the FMDV P1 protein, T cell specificities expand with each vaccine boost (Pedersen *et al*. 2016).

In conclusion, these correlations could carry potential for veterinary vaccine design, as SLA‐I Lr‐01.0 (6.28%), Lr‐04.0 (11.02%), Lr‐22.0 (6.74%) and Lr‐59.0 (6.19%) and SLA‐II Lr‐0.01 (4.55%), Lr‐0.15b (14.37%) and Lr‐0.27 (5.72%) are highly abundant haplotypes in European farmed pigs (Tables 1 & 2). On the other hand, targeting common haplotypes may reduce diversity over time, leading to susceptibility to other diseases and a lack of vaccine efficacy. Hence, a vaccine that works across a wide range of haplotypes potentially could be a safer strategy.

## Conﬂict of interest

The authors declare no known conﬂicts of interest associated with this publication.

## Supporting information

**Table S1** Detailed list of European farmed pigs incorporated in the present study.
**Table S2** Plate layout of the PCR primer panel for genotyping swine leukocyte antigen class I (a) and class II (b) alleles.
**Figure S1** Two basics concepts for swine leukocyte antigen haplotyping‐assisted animal trials in vaccine and transplantation research.
**Figure S2** Frequency of swine leukocyte antigen class I (a) and class II (b) low‐resolution haplotypes identified in 549 and 341 European farmed pigs by PCR screening assays respectively.
**Figure S3** Swine leukocyte antigen class I (a) and class II (b) low‐resolution haplotype diversity in nine and seven European commercial pig populations respectively.

## Data Availability

Further information about data and reagents used is available by request to the corresponding author. Minipig‐derived SLA typing data are confidential because of a non‐disclosure agreement.
