# Supplementary material for: Comparative analysis of swine leukocyte antigen gene diversity in European farmed pigs
Source: Anim Genet. 2021 May 24;52(4):523–31. doi: 10.1111/age.13090 (PMC8362188; doi:10.1111/age.13090)
Supplement: Supplementary file 1 — Table S1 Detailed list of European farmed pigs incorporated in the present study. Table S2 Plate layout of the PCR primer panel for genotyping swine leukocyte antigen class I (a) and class II (b) alleles. Figure S1 Two basics concepts for swine leukocyte antigen haplotyping‐assisted animal trials in vaccine and transplantation research. Figure S2 Frequency of swine leukocyte antigen class I (a) and class II (b) low‐resolution haplotypes identified in 549 and 341 European farmed pigs by PCR screening assays respectively. Figure S3 Swine leukocyte antigen class I (a) and class II (b) low‐resolution haplotype diversity in nine and seven European commercial pig populations respectively. [file AGE-52-523-s001.pdf]

Table S1 Detailed list of European farmed pigs incorporated in the present study.

		**no of pigs**		
**Breed**	**origin**	**SLA class I**	**SLA class II**	**Reference**
([LRxYS]xD)	DK	101	0	Pedersen *et al.* Vet. Immunol. Immunopathol. (2014) 162:108-116
	NL	8	8	Christine Jansen (2018, this study)
	UK	31	31	William T. Golde (2019-20, this study)
([LR×LW]×P)	Austria	9	9	Talker *et al.* Veterinary Research (2015) 46:52
		31	31	Talker *et al.* Journal of Virology (2016) 90:9364-9382
		8	8	Sabine Hammer (2012, this study)
LR x LW	UK	26	8	Franzoni *et al.* PLoS ONE (2013) 8:e84246
		10	10	Mokhtar *et al.* Frontiers in Immunology (2016) 7:40
	Spain	26	26	Burgara-Estrella *et al.* Viruses (2013) 5:663-677
		73	73	De León *et al.* Viruses (2020) 8:E513
YS x NL LR	UK	22	22	Mokhtar *et al.* Vaccine (2014) 32:6828-6837
YS x Ham	Sweden	44	0	Marianne Jensen-Waern (2015-16, this study)
YS		33	0	
GER LR	Germany	20	20	Ulrike Gimsa (2012, this study)
		65	65	Gimsa *et al.* Immunogenetics (2017) 69:39-47
AUT P	Austria	27	27	Essler *et al.* Animal Genetics (2013) 44:202-205
LW	UK	3	3	Simon Graham (2012, this study)
	CH	12	0	Artur Summerfield (2015, this study)
		**549**	**341**	**Total no of pigs**

LRYS/D, Animals were 25% Landrace (LR), 25% Yorkshire (YS), and 50% Duroc (D); LWLR/P, Animals were 25% Large White (LW), 25% Landrace (LR), and 50% Pietrain (P); LR × LW, Large White/Landrace crosses; YS × NL LR, Yorkshire/Dutch Landrace crosses; YS × Ham, Yorkshire/Hampshire crosses; GER LR, German Landrace; AUT P, Austrian Pietrain; LW, Large White.

Table S1 – References

Pedersen L.E., Jungersen G., Sorensen M.R., Ho C.-S. & Vadekær D.F. (2014) Swine leukocyte antigen (SLA) class I allele typing of Danish swine herds and identification of commonly occurring haplotypes using sequence specific low and high resolution primers. *Veterinary Immunology and Immunopathology* 162, 108-16. https://doi.org/10.1016/j.vetimm.2014.10.007

Talker S.C., Koinig H., Stadler M., Graage R., Klingler E., Ladinig A., Mair K.H., Hammer S.E., Dürrwald R., Ritzmann M., Saalmüller A. & Gerner W. (2015) Magnitude and kinetics of multifunctional $CD4^+$ and $CD8^+$ T cells in pigs infected with swine influenza A virus. *Veterinary Research* 46, 52-67. https://doi.org/10.1186/s13567-015-0182-3.

Talker S.C., Stadler M., Koinig H.C., Mair K.H., Rodriguez-Gomez I.M., Graage R., Zell R., Dürrwald R., Starick E., Harder T., Weissenböck H., Lamp B., Hammer S.E., Ladinig A., Saalmüller A. & Gerner W. (2016) Influenza A virus infection in pigs attracts multifunctional and cross-reactive T cells to the lung. *Journal of Virology* 90, 9364-82. https://doi.org/10.1128/JVI.01211-16.

Franzoni G., Kurkure N.V., Essler S.E., Everett H.E., Bodman-Smith K., Crooke H.R. & Graham S.P. (2013) Proteome-wide screening reveals immunodominance in the CD8 T cell response against classical swine fever virus with antigen-specificity dependant on MHC class I haplotype. *PLoS One* 8, e84246. https://doi.org/10.1371/journal.pone.0084246

Mokhtar H., Pedrera M., Frossard J.P., Biffar L., Choudhury B., Hammer S.E., Kvisgaard L.K., Larsen L.E., Stewart G.R., Somaravarapu S., Steinbach F. & Graham S.P. (2016) The non-structural protein 5 and matrix protein are major antigenic targets of T cell immunity to porcine reproductive and respiratory syndrome virus. *Frontiers in Immunology* 7, 40. https://doi.org/10.3389/fimmu.2016.00040.

Burgara-Estrella A., Díaz I., Rodríguez-Gómez I., Essler S.E., Hernández J. & Mateu E. (2013) Predicted peptides from non-structural proteins of porcine reproductive and respiratory syndrome virus are able to induce INF-γ and IL-10. *Viruses* 5, 663-677. https://doi.org/10.3390/v5020663

De León P., Cañas-Arranz R., Saez Y., Forner M., Defaus S., Bustos M.J., Torres E., Rodríguez-Pulido M., Andreu D., Blanco E., Sobrino F. & Hammer S.E. (2020) Association of porcine Swine Leukocyte Antigen (SLA) haplotypes with B- and T-cell immune response to foot-and-mouth disease virus (FMDV) peptides. Special issue: Evaluation of Vaccine Immunogenicity, *Vaccines (Basel)* 8, E513. https://doi.org/10.3390/vaccines8030513.

Mokhtar H., Eck M., Morgan S.B., Essler S.E., Frossard J.P., Ruggli N. & Graham S.P. (2014) Proteome-wide screening of the European porcine reproductive and respiratory syndrome virus reveals a broad range of T cell antigen reactivity. *Vaccine* 32, 6828-37. https://doi.org/10.1016/j.vaccine.2014.04.054

Gimsa U., Ho C.S. & Hammer S.E. (2017) Preferred SLA class I/class II haplotype combinations in German Landrace pigs. *Immunogenetics* 69, 39-47. https://doi.org/10.1007/s00251-016-0946-6.

Essler S.E., Ertl W., Deutsch J., Ruetgen B.C., Groiss S., Stadler M., Wysoudil B., Gerner W., Ho C.-S. & Saalmüller A. (2013) Molecular characterization of swine leukocyte antigen gene diversity in purebred Pietrain pigs. *Animal Genetics* 44, 202-205. https://doi.org/10.1111/j.1365-2052.2012.02375.x

Table S2a Plate layout of the PCR primer panel for genotyping swine leukocyte antigen (SLA) class I alleles.

	H	G	F	E	D	C	B	A
1	**Negative Control**	**209 bp** SLA-1*01XX(all)	**147 bp** SLA-1*02XX(all)	**181 bp** SLA-1*04XX(all)	**220 bp** SLA-1*05XX(all); SLA-1*16:02	**163 bp** SLA-1*06XX(all); SLA-1*13:01	**220 bp** SLA-1*07XX(all)	**138 bp** SLA-1*08XX(all)
2	**195 bp** SLA-1*09XX(all)	**180 bp** SLA-1*10XX(all)	**182 bp** SLA-1*11XX(all)	**119 bp** SLA-1*12XX(all)	**211 bp** SLA-1*13XX(all)	**219 bp** SLA-1*14XX(all)	**253 bp** SLA-1*15XX(all); SLA-1*14:02; SLA-2*01XX(all)	**173 bp** SLA-1*16XX(all)
3	**134 bp** SLA-1*16XX(all); SLA-1*16:02; SLA-2*03XX(all); SLA-2*11:04	**208 bp** SLA-1*17:01	**193 bp** SLA-1*07:03; SLA-2*11:04	**130 bp** SLA-1*07:03; SLA-1*07:05	**196 bp** SLA-1*18:01	**177 bp** SLA-3*01XX(all)	**183 bp** SLA-3*03XX(all); SLA-3*08XX(all)	**192 bp** SLA-3*04XX(all); SLA-3*04:04 SLA-2*15XX(all)
4	**138 bp** SLA-3*05XX(all)	**187 bp** SLA-3*06XX(all); SLA-3*07XX(all)	**152 bp** SLA-3*07XX(all)	**152 bp** SLA-3*06:01	**139 bp** SLA-3*06:02	**139 bp** SLA-1*11:03; SLA-3*04XX(all); 04:04 SLA-3*03XX excl. 03:06	**172 bp** SLA-2*01XX(all)	**138 bp** SLA-2*02XX(all)
5	**89 bp** SLA-2*03XX(all)	**311 bp** SLA-2*04XX(all)	**127 bp** SLA-2*05XX(all)	**125 bp** SLA-2*06XX(all)	**199 bp** SLA-2*07XX(all)	**126 bp** SLA-2*08XX(all)	**177 bp** SLA-2*09XX(all)	**104 bp** SLA-2*10XX(all)
6	**123 bp** SLA-2*11XX(all)	**160 bp** SLA-1*14:02; SLA-2*12XX(all)	**117 bp** SLA-1*11:03; SLA-2*13XX(all)	**131 bp** SLA-1*09XX(all); SLA-2*14XX(all) 16XX(all); 16:02	**90 bp** SLA-2*15XX(all); SLA-2*11:04	**196 bp** SLA-2*06:01~02 / 06:06; 09:01/09:02; 16XX(all)	**138 bp** SLA-1*07XX(all) SLA-2*02XX(all) SLA-2*16XX(all); SLA-2*17:01	**175 bp** SLA-2*16:02

Reference: Ho *et al.* Animal Genetics (2009) 40:468-78. https://doi.org/10.1111/j.1365-2052.2009.01860.x.

Table S2b Plate layout of the PCR primer panel for genotyping swine leukocyte antigen (SLA) class II alleles.

	H	G	F	E	D	C	B	A
7	**Negative**	**162 bp**	**203 bp**	**115 bp**	**180 bp**	**206 bp**	**172 bp**	**122 bp**
	**Control**	DRB1*01XX(all)	DRB1*01XX(all)	DRB1*02XX(all)	DRB1*03XX(all)	DRB1*04XX(all)	DRB1*05XX(all)	DRB1*06XX(all)
		DRB1*13:02/01:03	DRB1*13:02					
		DRB1*01:04/07:04	DRB1*01:04/01:05					
8	**133 bp**	**108 bp**	**105 bp**	**157 bp**	**135 bp**	**109 bp**	**186 bp**	**182 bp**
	DRB1*07XX(all)	DRB1*08XX(all)	DRB1*09XX(all)	DRB1*09XX(all)	DRB1*10XX(all)	DRB1*11XX(all)	DRB1*12XX(all)	DRB1*13XX(all)
		DRB1*03:02	DRB1*09:05/09:06	DRB1*16:02/17:01	DRB1*10:06/10:07			
		DRB1*16:01/16:03	DRB1*09:04	DRB1*09:06/09:07				
9	**113 bp**	**160 bp**	**134 bp**	**202 bp**	**197 bp**	**117 bp**	**160 bp**	**118 bp**
	DRB1*14XX(all)	DRB1*14:02	DRB1*15:01	DRB1*17:01	DRB1*10:07/10:08	DRB1*04:03~04	DRB1*04:01~02	DRB1*04:01~02
				DRB1*15:02	DRB1*09:07	DRB1*11:01/11:03	DRB1*04:05/04:06	DRB1*04:05/04:06
				DRB1*15:03		DRB1*14:02		
10	**165 bp**	**180 bp**	**146 bp**	**166 bp**	**197 bp**	**193 bp**	**204 bp**	**154 bp**
	DQB1*01XX(all)	DQB1*01XX(all)	DQB1*02XX(all)	DQB1*03XX(all)	DQB1*04XX(all)	DQB1*05XX(all)	DQB1*06XX(all)	DQB1*07XX(all)
		DQB1*11:01					DQB1*06:03	
11	**148 bp**	**146 bp**	**180 bp**	**161 bp**	**193 bp**	**176 bp**	**133 bp**	**165 bp**
	DQB1*08XX(all)	DQB1*09XX(all)	DQB1*09XX(all)	DQB1*10:01	DQB1*02XX	DQB1*02:02 / 02:06	DQB1*02XX	DQB1*02:03/02:06
	DQB1*08:06		DQB1*03:04	DQB1*10:02	DQB1*03:04/08:06			DQB1*03:01
12	**173 bp**	**141 bp**	**210 bp**	**160 bp**	**124 bp**	**148 bp**	**111 bp**	**120 bp**
	DQB1*02:02/02:04	DQA*01XX(all)	DQA*02XX(all)	DQA*03XX(all)	DQA*04XX(all)	DQA*04XX(all)	DQA*05XX(all)	DQA*06:01
	DQB1*08:06		DQA*06:01					

Reference: Ho *et al.* Animal Genetics (2010) 41:428-32. https://doi.org/10.1111/j.1365-2052.2010.02019.x.

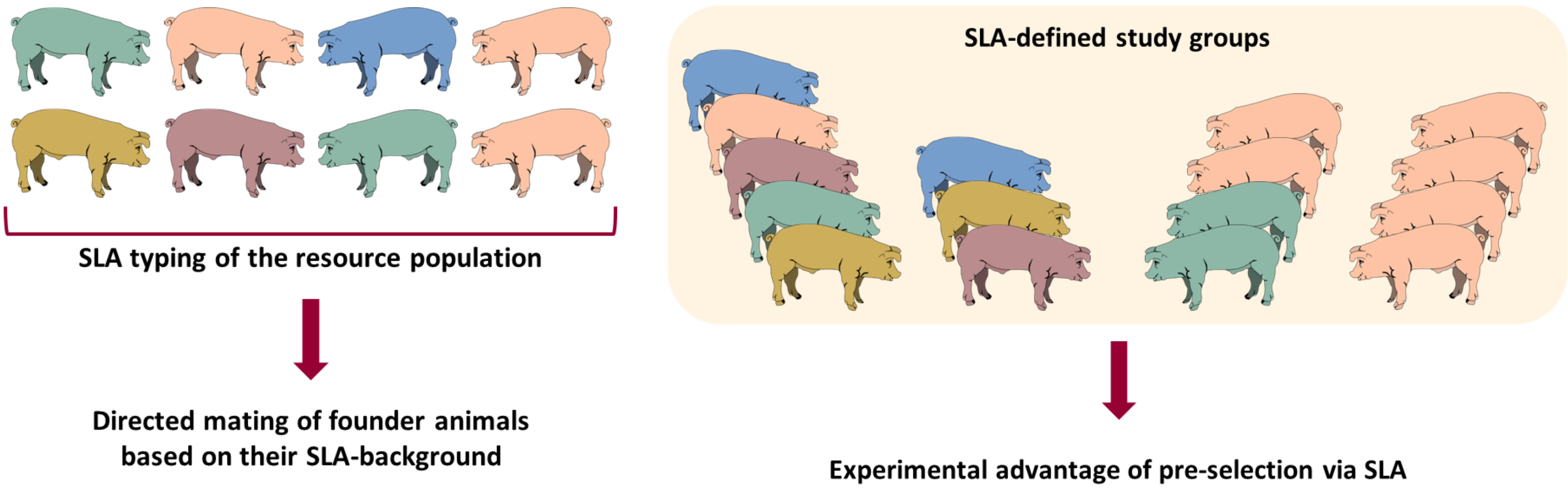

Figure S1 Two basics concepts for Swine leukocyte antigen (SLA) haplotyping-assisted animal trials in vaccine and transplantation research.

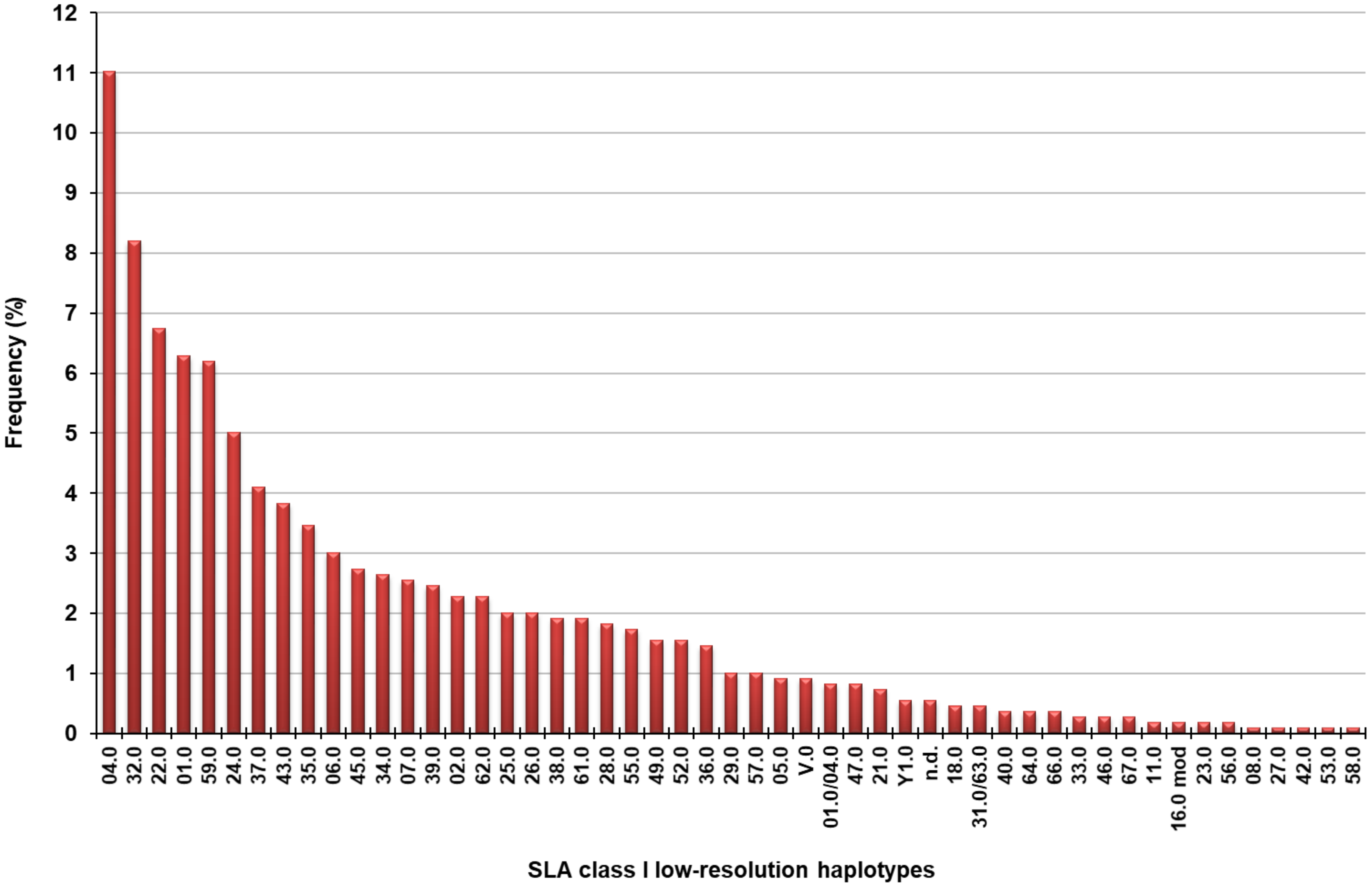

Figure S2a Frequency of swine leukocyte antigen (SLA) class I low-resolution haplotypes identified in 549 European farmed pigs.

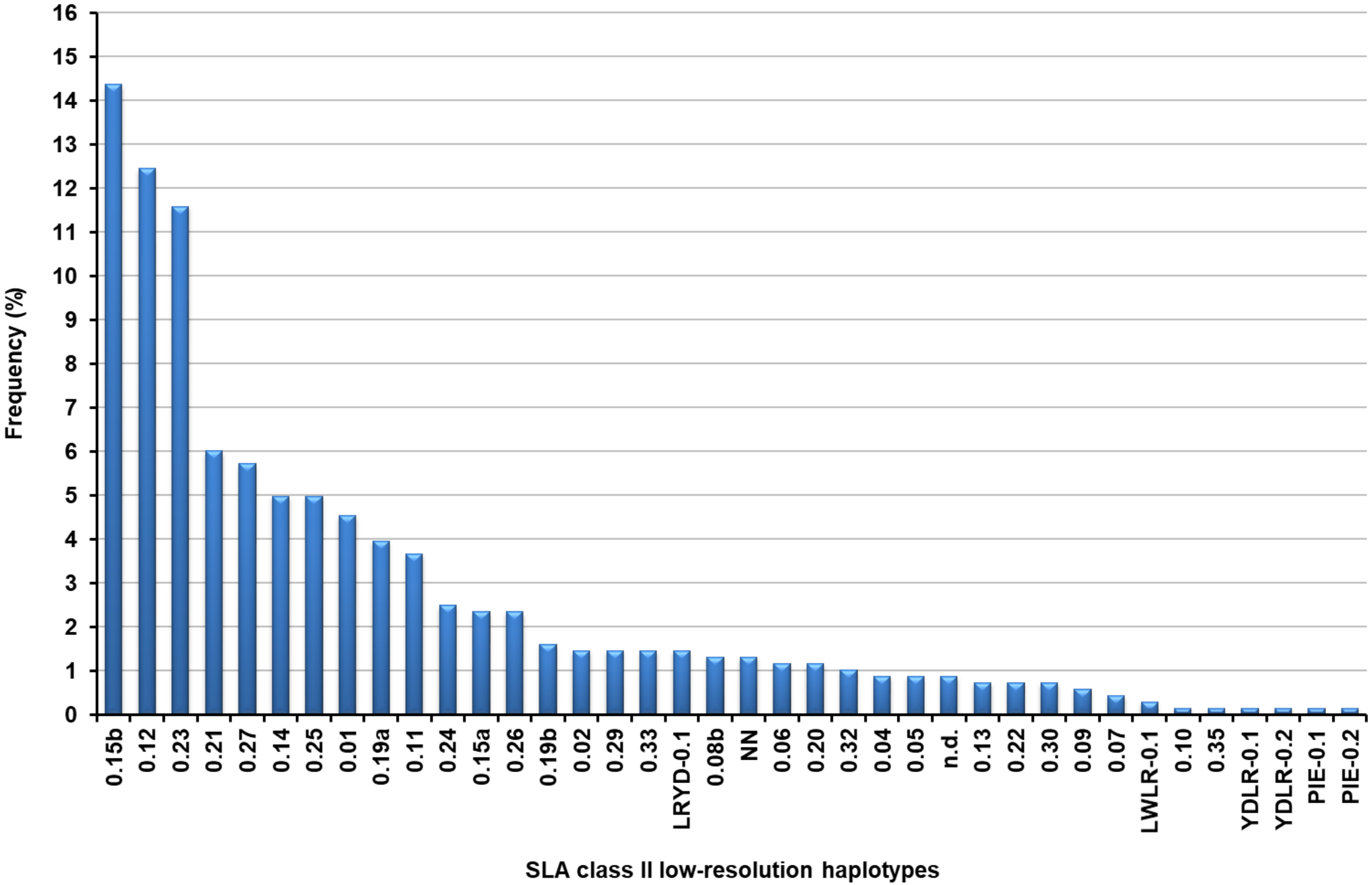

Figure S2b Frequency of swine leukocyte antigen (SLA) class II low-resolution haplotypes identified in 341 European farmed pigs.

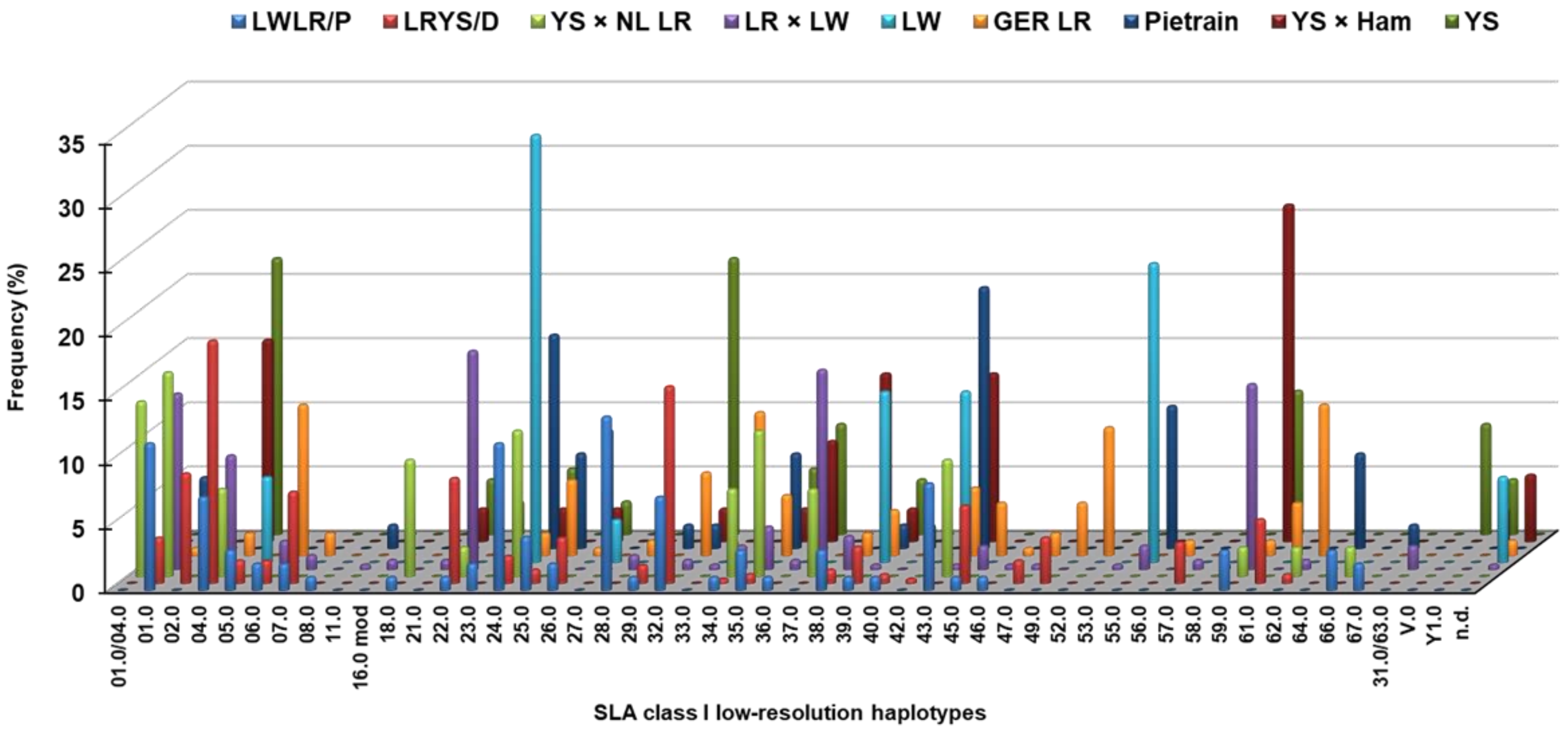

Figure S3a Swine leukocyte antigen (SLA) class I low-resolution haplotype diversity in nine European commercial pig populations. LWLR/P, Animals were 25% Large White (LW), 25% Landrace (LR), and 50% Pietrain (P); LRYS/D, Animals were 25% Landrace (LR), 25% Yorkshire (YS), and 50% Duroc (D); YS × NL LR, Yorkshire/ Dutch Landrace crosses; LR × LW, Large White/Landrace crosses; GER LR, German Landrace; YS × Ham, Yorkshire/Hampshire crosses.

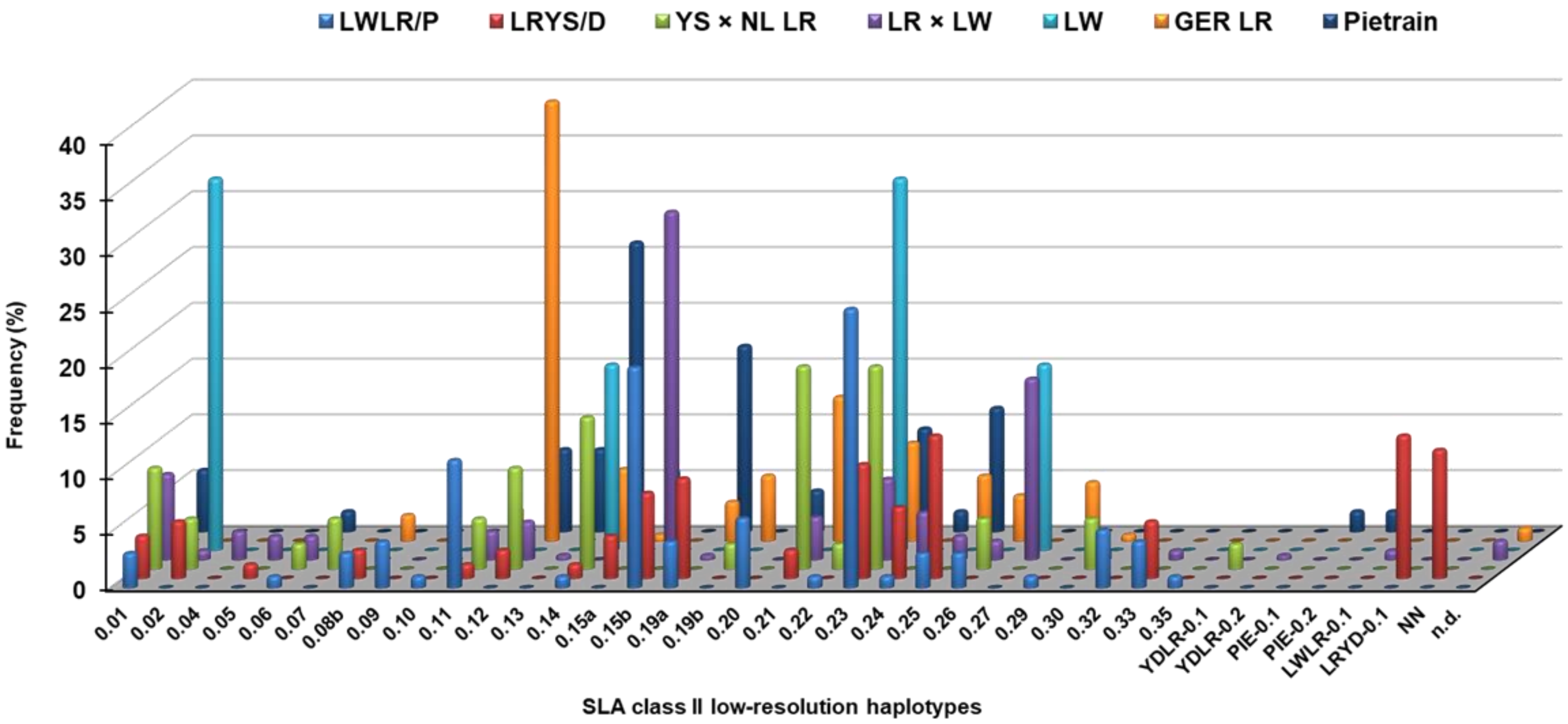

Figure S3b Swine leukocyte antigen (SLA) class II low-resolution haplotype diversity in seven European commercial pig populations. LWLR/P, Animals were 25% Large White (LW), 25% Landrace (LR), and 50% Pietrain (P); LRYS/D, Animals were 25% Landrace (LR), 25% Yorkshire (YS), and 50% Duroc (D); YS × NL LR, Yorkshire/ Dutch Landrace crosses; LR × LW, Large White/Landrace crosses; GER LR, German Landrace.
